# The miR-200 family is increased in dysplastic lesions in ulcerative colitis patients

**DOI:** 10.1371/journal.pone.0173664

**Published:** 2017-03-13

**Authors:** Amy Lewis, Carla Felice, Tomoko Kumagai, Cecilia Lai, Kriti Singh, Rosemary R. Jeffery, Roger Feakins, Eleni Giannoulatou, Alessandro Armuzzi, Noor Jawad, James O. Lindsay, Andrew Silver

**Affiliations:** 1 Centre for Genomics and Child Health, Blizard Institute, Barts and The London School of Medicine & Dentistry, London E1 2AT United Kingdom; 2 IBD Unit, Complesso Integrato Columbus, Gemelli Hospital Catholic University Foundation, Rome, Italy; 3 Department of Histopathology, The Royal London Hospital, London, E1 1BB United Kingdom; 4 Victor Chang Cardiac Research Institute, Darlinghurst, New South Wales, Australia; 5 The University of New South Wales, Sydney, New South Wales, Australia; 6 Barts Cancer Institute, Barts and The London School of Medicine & Dentistry, London EC1M 6BQ United Kingdom; 7 Centre for Immunobiology, Blizard Institute, Barts and The London School of Medicine & Dentistry, London E1 2AT United Kingdom; University of South Alabama Mitchell Cancer Institute, UNITED STATES

## Abstract

**Background:**

Colorectal cancer (CRC) is a life-threatening complication of ulcerative colitis (UC), and patients are routinely screened for the development of precancerous lesions (dysplasia). However, rates of CRC development in patients with confirmed low-grade dysplasia vary widely between studies, suggesting a large degree of heterogeneity between these lesions that is not detectable macroscopically. A better understanding of the underlying molecular changes that occur in dysplasia will help to identify lesions at higher risk of malignancy. MicroRNAs (miRNAs) post-transcriptionally regulate protein expression and cell-signalling networks. Aberrant miRNA expression is a feature of sporadic CRC but much less is known about the changes that occur in dysplasia and in UC.

**Methods:**

Comprehensive microRNA profiling was performed on RNA extracted from UC dysplastic lesions (n = 7) and UC controls (n = 10). The expression of miRNAs in UC post inflammatory polyps (n = 7) was also assessed. Candidate miRNAs were further validated by qPCR, and miRNA *in situ* hybridization. Serum levels of miRNAs were also assessed with a view to identification of non-invasive biomarkers of dysplasia.

**Results:**

UC dysplasia was associated with a shift in miRNA expression profiles that was not seen in inflammatory polyps. In particular, levels of miR-200b-3p were increased in dysplasia, and this miRNA was localised to epithelial cells in dysplastic lesions and in UC cancers. No changes in miRNA levels were detected in the serum.

**Conclusion:**

UC-Dysplasia is linked to altered miRNA expression in the mucosa and elevated miR-200b-3p levels.

## Introduction

Inflammatory bowel diseases (IBD), which include ulcerative colitis (UC), are characterised by uncontrolled intestinal inflammation. Colorectal cancer (CRC) is a life-threatening complication of UC, and UC patients are estimated to be at a 2.4 fold increased risk of CRC relative to the general population.[1] The risk of CRC in UC patients increases considerably with duration and extent of active inflammation, diagnosis at a younger age, concomitant primary sclerosing cholangitis (PSC) and family history of CRC.[2,3] Relative to sporadic CRC, UC associated CRC (UC-CRC) is linked to an earlier age of onset and higher rates of mortality; in one study, 59% of UC-CRC patients had died at 5-years of follow-up.[3]

Given the increased risk of UC-CRC, it is necessary to screen UC patients for the development of precancerous lesions (dysplasia), with periodic chromoendoscopies starting 8–10 years after the first appearance of colitis-associated symptoms.[4,5] During surveillance procedures, targeted biopsies are collected from suspect lesions and assessed by pathologists. The presence of dysplasia is then used to guide and inform the subsequent clinical management of the patient: for low-grade dysplasia (LGD) on flat mucosa it is advisable to reduce the interval between surveillance colonoscopies, in the case of high-grade dysplasia (HGD) colectomy is indicated.[4,5]

UC-associated post-inflammatory polyps (UC-IPs) (pseudopolyps) are raised areas of inflamed mucosa or granulation tissue seen after intestinal mucosal recovery from inflammatory damage. A history of UC-IP may be an indication of the severity of prior inflammation. Current endoscopic surveillance programmes do not consider UC-IPs *per se* important clinically and their recording may well be incomplete. Yet, a correlation between the presence of colonic UC-IPs and increased risk of CRC has been proposed.[6–8] However, it remains to be determined whether the link between UC-IPs and CRC is indirect or whether UC-IPs show any features or changes in gene expression that might be associated with an increased risk of malignancy.

The molecular mechanisms that lead to cancer from LGD in UC are poorly understood, and rates of UC-CRC occurrence in patients with diagnosed LGD vary widely between studies 0–54%;[9–14] current estimates are that 19% of patients with a diagnosis of LGD develop HGD or cancer.[15] This variation in outcomes probably reflects the fact that although LGD is treated as a single entity it encompasses a range of underlying molecular changes which cannot be distinguished macroscopically or histologically. Hence there is a significant clinical need for molecular biomarkers to identify those dysplastic lesions that are at high risk of neoplastic progression and malignancy.

MicroRNAs (miRNAs) are small non-coding RNAs that inhibit protein translation by binding to complementary sequences in the 3’UTR of target mRNAs. Each miRNA is predicted to bind to multiple potential mRNAs targets and thereby alter signalling pathways within cells. MiRNAs have important roles in development and disease and have attracted particular attention for their role in cancer and IBD, and for their potential use as biomarkers and therapeutic targets.[16] A role for miRNAs in sporadic CRC has been established.[17] However, far less is known about their function in UC-CRC and dysplasia with only two studies reported assessing patients with both UC and CD.[18,19] The techniques used and the clinical characteristics of the specimens analysed also differ between studies. Moreover, because of the low frequency of IBD-dysplasia the cohorts analysed are relatively small and the result requires independent validation.[18,19]

In order to address the role of miRNAs in UC-dysplasia, we have determined miRNA expression in a cohort of UC patients with and without dysplasia, using samples collected from IBD surveillance centres across the UK. We have also compared miRNAs identified in our study of UC with findings in the published literature on IBD-dysplasia. In addition, we provide the first comprehensive analysis of miRNAs in UC-IPs and compare changes in miRNA profiles between these polyps and UC controls. As miRNAs are also exported in the wider circulation, the potential to utilise miRNAs as non-invasive biomarkers of UC-Dysplasia (UC-DYS) was also explored in a proof-of-principle study.

## Materials and methods

### Ethics statement and patient selection

Ethics were obtained to recruit patients from multiple hospitals in UK (East London and The City Research Ethics Committee 1, REC 09/H0703/116; UKCRN ID 8099). Inclusion criteria in the study included a diagnosis of UC and enrolment in a surveillance programme. Patients gave written informed consent on forms approved by the Ethics committee and were recruited between 2010 and 2014.

The clinical and endoscopic disease activity was recorded for each patient, using the Mayo score.[20] UC phenotype was classified according to Montreal criteria.[21] Further clinical characteristics were also recorded, including age, ethnicity, disease duration, smoking habit, medications, adherence to therapy, family history of colon cancer, concomitant diseases and previous history of dysplasia.

### Collection and selection of samples for microRNA analysis

Multiple biopsies were collected from 5 sites throughout the bowel during routine endoscopy for histopathology and miRNA analysis. Biopsies for miRNA analysis were stored in RNAlater at -80°. The local pathologist assessed biopsies taken for histopathology. The resulting pathology reports were used to classify the biopsies taken from the same site and stored in RNAlater into those with and without dysplasia, and with and without evidence of histological inflammation, (neutrophil infiltration of the epithelium). A peripheral blood sample was also collected and centrifuged to obtain serum, and stored at -80°C until further experiments.

To reduce possible confounding factors, samples from patients with coexisting primary sclerosing cholangitis, previous history of dysplasia or CRC and colitis limited to the left-sided colon or to the rectum were excluded from this study. Those samples used here were from patients with pancolitis (E3 Montreal).

### Tissue miRNA arrays

Biopsies stored in RNA later were homogenised in 700μL Qiazol, and RNA extractions were performed using the miRNAesy Kit (Qiagen, UK) as per the manufacturer’s instructions. The resultant RNA was sent to Exiqon (Denmark) for miRNA profiling using the miRCURY LNA™ microRNA Array (7th Gen). The quantified signals were background corrected and normalized using the quantile normalization method. The normalised data was then log2 transformed prior to statistical analysis.

### Serum miRNA arrays

Serum samples were sent to Exiqon (Denmark) and RNA extracted and analysed by qPCR using miRCURY LNATM Universal RT microRNA PCR Human panel I+II. Samples were run in two batches, and each batch contained UC controls and UC-dysplasia samples (batch one: n = 5 and n = 3, respectively; batch two: n = 5 and n = 5, respectively). The data normalisation was performed based on the average of the assays detected in all samples in each batch respectively, and the normalised data log2 transformed. The data for each batch was then median-centred and combined prior to statistical analysis to account for ‘batch’ effects. Finally, miRNAs were filtered to include only miRNAs that were detected above the background threshold in all samples tested. The aim was to ensure that only robustly expressed miRNAs were considered.

### MiRNA qPCR validation

A select number of miRNAs were evaluated by qPCR using the miScript primer assays (S1 Table). For qPCR analysis 1000 ng of RNA was reversed transcribed using the miScript II RT Kit (Qiagen) and the miScript HighSpec buffer. The resultant cDNA was then diluted 10-fold prior to qPCR analysis using the miScript miRNA quantification system (Qiagen). Each reaction was performed in duplicate and melt curves performed to ensure a single PCR product was synthesised. The resultant cycle threshold values (Ct’s) were exported into Excel, duplicates were averaged, and data normalised to the geometric mean of RNU6 and SNORD42b, two stably expressed small non-coding RNAs. The normalised expression values were then log2 transformed to equalise variance prior to statistical analysis.

### microRNA in situ hybridization

The levels of miR-200b-3p and miR-21-5p were investigated using miRNA *in situ* hybridization (ISH) technology (Exiqon, Denmark) in formalin fixed paraffin embedded (FFPE) tissue taken from the colon of UC patients. All blocks were obtained from the Royal London Pathology archives, and 4 μm sections were cut under RNAse-free conditions. Prior to staining sections were baked at 65°C for 10 mins before deparaffinising in xylene for 10 mins and hydrating through graded alcohols to RNAse-free H_2_O. Sections were digested with 10ug/ml proteinase K in PBS at 37°C for 30 minutes, washed in RNAse-free H_2_O and dehydrated. Sections were then incubated with Double-DIG (digoxigenin) labelled Locked Nucleic Acid (LNA) ISH probes (60 nM, Exiqon) for miR-200b-3p (619853–360), miR-21-5p (619870–360) or a scrambled negative control (699004–360) at 56°C for 2 hours before a series of stringency washes and staining performed as given previously.[22] A clinical pathologist [RF] at the Royal London Hospital confirmed positive staining and reviewed all slides.

### Statistical analyses

Unsupervised principal component analysis (PCA) of miRNA levels in UC controls, UC-DYS and UC-IPs was performed to explore natural grouping in the data using Solo software (Eigenvector, USA). The software’s proprietary algorithms were used to exclude miRNAs with excessive missing values and replace any further missing values in the remaining data. The PCA was carried out on the covariance matrix using the log2 transformed array data. Three components were fitted to the model and these were sufficient to separate UC controls from UC dysplasia.

To identify individual differentially expressed miRNAs, the array data (both tissue and serum) were analysed using the ‘Significance Analysis of Microarrays’ package in R. Missing values were compensated for using the K-Nearest Neighbours Imputer algorithm, and the number of neighbours set to 6. The log2 transformed data were median-centred, and a two-class unpaired analysis was performed with 1000 permutations. A significance cut-off was set using a delta value of 0.5 and a 2-fold minimum change. Under these stringent cut-offs the estimated false discovery rate was 0.00%.

For the qPCR validation analysis a two-way analysis of variance (ANOVA) test was used to explore the relationship between miRNAs in dysplasia and inflammation, each modelled as independent discrete factors. *Post-hoc* analyses were then performed to identify difference between groups with alpha adjusted for multiple testing using the Bonferroni criterion.

### In silico identification of microRNA targets

Experimentally validated target genes for candidate miRNAs were obtained using miRTarBase,[23] which contains targets that have been validated by reporter assays, western blot, qPCR, microarray and next-generation sequencing experiments. miRNA-target interactions were also investigated based on the collective information of functional studies of miRNAs in the database. To bioinformatically predict target genes based on the miRNA seed sequence, two predictive algorithms were used, TargetScan as well as miRDB.[24,25]

To investigate the influence of candidate miRNA transcript levels in UC dysplasia, we interrogated the gene signatures obtained by cDNA microarrays in 4 UC patients without dysplasia and 11 UC patients harbouring remote neoplasia.[26] To obtain a list of all differentially expressed genes across the two groups, the microarray datasets were obtained from the GEO repository (accession number GSE37283) and analysed with GEO2R, a web-based application that performs comparisons between groups on the original submitter-supplied microarray datasets. GEO2R uses the GEOquery and limma R packages from the Bioconductor project. For consistency, to identify differentially expressed genes, we applied the following thresholds: adjusted p-value < 0.05 (based on the Benjamini & Hochberg false discovery rate method) and |log2(fold-change)| > 1.

## Results

### Study design and biopsy characteristic

To interrogate miRNA profiles in UC-DYS lesions, RNA extracted from biopsies collected during routine surveillance endoscopies with and without evidence of dysplasia was analysed (n = 10 and n = 7, UC controls and UC-DYS, respectively); the clinical characteristics of patients from whom a UC dysplasia sample was taken is given in Table 1. For the array, the cohort was predominantly White (6/7), the median age was 73 years, and all patients had a history of pancolitis (E3 disease). Biopsies were taken from sites of dysplasia throughout the colon: 2 from the ascending colon, 2 from the transverse colon, 2 from sigmoid colon and 1 from the rectum. Of these, 6/7 (86%) was classified as LGD and 1/7 had HGD; 3/7 biopsies also had evidence of histological inflammation. RNA isolated from UC-IPs (n = 7) was also analysed (S2 Table).

**Table 1:** Characteristics of ulcerative colitic patients from whom a dysplasia specimen was obtained. All were from patients classified as Montreal E3.

Pt	Gender	Age	Ethnicity	Disease duration (years)	Smoking habit	Dysplasia site	Active (Y/N)	5ASA (Y/N)	Tissue analysed by array
1	F	73	White	42	Never	Sigmoid	Y	Y	Y
2	M	75	Indian	28	Never	Transv	N	Y	Y
3	F	55	White	20	Never	Ascend	Y	Y	Y
4	F	59	White	15	Never	Transv	Y	Y	Y
5	F	73	White	34	Never	Rectum	N	Y	Y
6	F	62	White	30	Never	Sigmoid	N	Y	Y
7	M	81	White	32	Former	Ascend	N	Y	Y
8	M	69	White	32	Never	Transv	Y	—	N
9	M	50	White	37	Never	Caecum	Y	Y	N
10	M	56	Bangladeshi	5	Never	Transv & Caecum	Y	Y	N

To identify miRNAs associated with UC-DYS that were independent of intestinal location and disease activity, control biopsies were pooled from a number of UC patients with respect to presence or absence of histological inflammation (active and inactive) and the five-biopsy sites (ascending colon, transverse colon, descending colon, caecum, or rectum). This gave a final 10 control samples. RNA isolated from UC-IPs (n = 7) was also analysed to determine whether these possessed similar changes in gene expression to dysplasia specimens.

### Identification of miRNAs associated with dysplasia in ulcerative colitis

**Fig 1:**
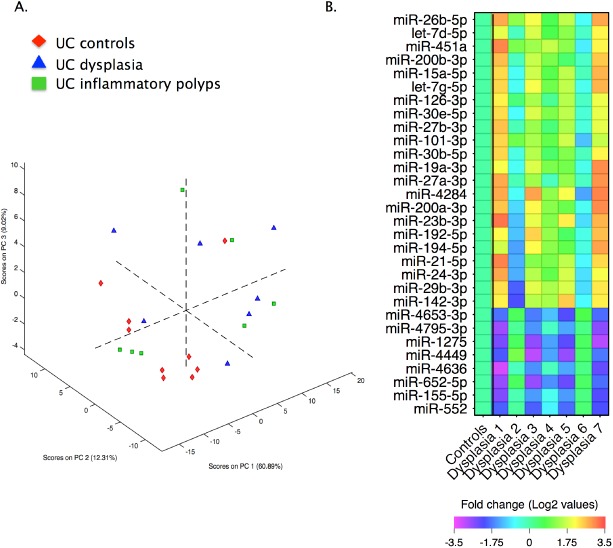
Altered miRNA profiles in ulcerative colitis associated-dysplasia. (A) Principal component analysis of UC-controls, UC-Dysplasias and UC-inflammatory polyps, showing natural groupings within the data along the first three principal components. (B) Heatmap of miRNAs identified by permutation analysis as differentially expressed in UC-dysplasia relative to UC-controls. The data are presented as Log2 fold change in UC-dysplasia relative to UC-controls, and the y-axis is ranked accordingly.

The expression levels of 1899 miRNAs were assessed by array, of which 1240 (65%) were not detected above the background threshold levels in any of the 24 samples analysed and therefore excluded. One control sample (an active biopsy from the caecum) was further excluded on the basis of a lower call rate than the remaining samples (S1 Fig). To explore natural grouping within the data unsupervised principal component analysis (PCA) was performed. For PCA, miRNAs with excessive missing values were excluded (a total of 258), and only the remaining 401 miRNAs were considered (S1 Fig). The resultant PCA model demonstrated some separation of UC-DYS from control samples, suggesting that UC-DYS is associated with a shift in miRNA expression profiles. Control samples were separated completely from dysplasia samples within the first three principal components with the sole exception of one sample, an active rectal biopsy (Fig 1A).

To identify differentially expressed individual miRNAs the array data was analysed using ‘Significance Analysis of Microarrays’ software with an estimated false discovery rate of 0% and a fold change >2 set as stringent cut-off values for significance. This analysis identified 22 miRNAs that were upregulated in dysplasia and 8 miRNAs that were present at lower levels in UC-DYS lesions (Table 2; Fig 1B). A number of the miRNAs identified had been previously linked to IBD-DYS, including miR-200b-3p, miR-200a-3p, miR-192-5p, and miR-155-5p13. Previously reported associations between miR-21-5p12 were also confirmed (S2B Fig); reported difference in miR-31 in IBD-dysplasia[19] could not be fully assessed here as miR-31-5p expression was only detected in 2 of the 24 samples analysed by array. However, it is noteworthy that both biopsies where miR-31-5p levels were detected above the background threshold were dysplastic (S2D Fig).

**Table 2:** miRNAs associated with ulcerative colitis-dysplasia.

miRNA ID	Fold Change in dysplasia	q-value	Links to IBD dysplasia
miR-26b-5p	2.8789	<0.001	
let-7d-5p	2.1092	<0.001	
miR-451a	2.6647	<0.001	
miR-200b-3p	2.2278	<0.001	
miR-15a-5p	2.3591	<0.001	Up regulated in IBD-dysplasia[19]
let-7g-5p	2.3972	<0.001	
miR-126-3p	2.1196	<0.001	
miR-30e-5p	2.2123	<0.001	
miR-27b-3p	2.063	<0.001	
miR-101-3p	2.2401	<0.001	
miR-30b-5p	2.0575	<0.001	
miR-19a-3p	2.2133	<0.001	
miR-27a-3p	2.2084	<0.001	
miR-4284	2.7182	<0.001	
miR-200a-3p	2.3164	<0.001	Up regulated in IBD-dysplasia[19]
miR-23b-3p	2.5008	<0.001	
miR-192-5p	2.1055	<0.001	Up regulated in IBD-dysplasia[19]
miR-194-5p	2.0351	<0.001	Up regulated in IBD-dysplasia[19]
miR-21-5p	2.0956	<0.001	Up regulated in IBD-dysplasia[18]
miR-24-3p	2.0126	<0.001	
miR-29b-3p	2.0203	<0.001	
miR-142-3p	2.0688	<0.001	Down regulated in IBD-dysplasia[19]
miR-552	0.4573	<0.001	Up regulated in IBD-dysplasia[19]
miR-155-5p	0.4607	<0.001	Down regulated in IBD-dysplasia[19]
miR-652-5p	0.3789	<0.001	
miR-4636	0.4386	<0.001	
miR-4449	0.4066	<0.001	
miR-1275	0.3564	<0.001	
miR-4795-3p	0.4577	<0.001	
miR-4653-3p	0.4885	<0.001	

### Changes in miRNA profiles in post inflammatory polyps

To determine whether the miRNA profiles of UC-IPs resembled UC-DYS specimens or not, the UC-IP miRNA expression changes were fitted to the PCA model. Interestingly, UC-IPs appeared to form an intermediate group, with some samples clustering with controls and other samples with the UC-DYS specimens (Fig 1A). However, relative to controls, in terms of absolute differences in expression, there were only two miRNAs with a fold change>2 at a FDR of 0.00% in UC-IPs using the SAM software: miR-144-3p was increased in UC-IPs (2.017 fold, q<0.001) and miR-4795-3p was reduced (0.456 fold, q<0.001). There was no overlap between miRNAs that marked UC-IPs and those that were associated with UC-DYS.

### qPCR validation of miRNA changes in dysplasia

To confirm the findings of the array, qPCR validation was performed for 7 miRNAs upregulated and 1 miRNAs down-regulated in UC-DYS in the array. The validation cohort was expanded in number, but included samples previously analysed by array and therefore does not represent a wholly independent cohort (UC-DYS n = 10 and UC controls n = 16); where multiple biopsies were taken from individual control UC patients they were averaged with respect to disease activity to avoid pseudo-replication of the data. There were no significant differences between UC-DYS and UC controls in our initial analysis (t-test, S3 Table).

**Fig 2:**
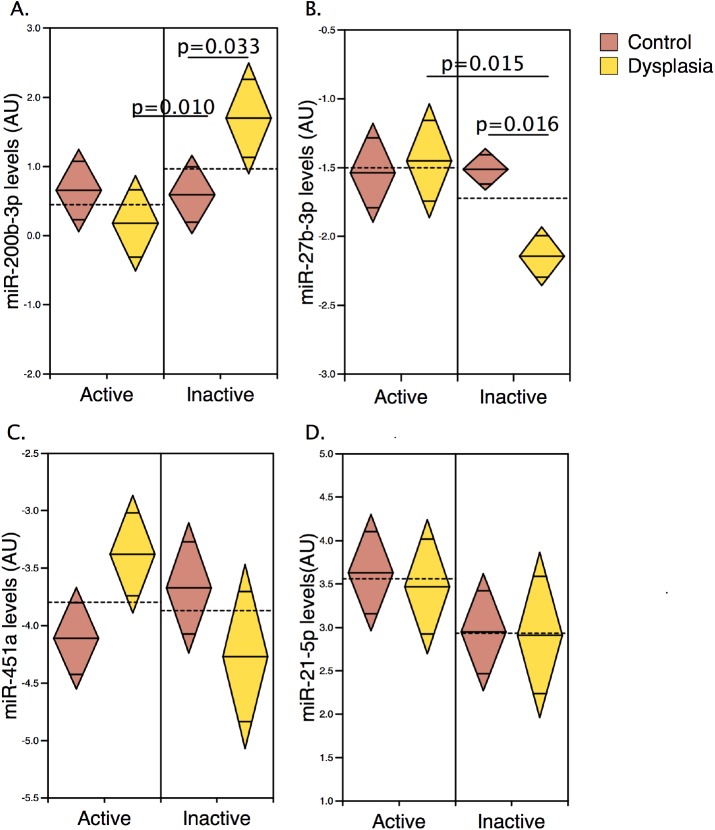
qPCR validation of miRNAs associated with ulcerative colitis-dysplasia. qPCR validation of miRNAs associated with UC-DYS. (A-D) qPCR analysis of miR-200b-3p, miR-27b-3p, miR-451a and miR-21-5p expression levels, in an expanded validation UC cohort. Significant difference were determined by two-ANOVA and the data are presented as a two-way diamond mean comparison plot. The horizontal dashed line represents the overall mean. The line through the centre of each diamond corresponds to the individual groups mean. The top and bottom diamond vertices indicate the respective upper and lower 95% confidence limits of the group means.

One potential confounding factor in this analysis is a change in the proportions of UC-DYS samples with active disease analysed in the validation cohort compared to the array cohort: 3/4 in the array cohort vs. 7/11 in the validation cohort. Hence a two-way ANOVA was performed to investigate the relationship between dysplasia, disease activity and miRNAs levels, followed by *post-hoc* comparisons of groups. This demonstrated that for 3 of the miRNAs tested (miR-200b-3p, miR-451a, and miR-27b), there was a significant interaction term for UC-DYS and disease activity (Table 3). A subsequent *post-hoc* test demonstrated that miR-200b-3p was significantly up-regulated (fold change >2) in UC-DYS specimens in the absence of histological inflammation relative to inactive controls in line with the results of the array (p = 0.033). However, as disease activity tended to supress miR-200b-3p levels in the UC-DYS group (p = 0.010), dysplasia–associated differences in expression were not seen on a background of histological inflammation (Fig 2A). Conversely, and in contrast to the array, a significant reduction in miR-27b was observed in UC-DYS relative to controls on an inactive background (p = 0.016); in the UC-DYS group histological inflammation was associated with higher miR-27b levels (p = 0.015) (Fig 2B). No significant differences in miR-451a were identified in the *post-hoc* comparisons of the groups once alpha was adjusted for multiple testing (Fig 2C). In contrast to previous studies miR-21-5p showed a stronger association with disease activity than with dysplasia (Fig 2D).

**Table 3:** Two-way analysis of variance of miRNA levels in ulcerative colitis-associated dysplasia and controls.

miRNA ID	Two-way ANOVA			*Posthoc* comparisons			
	Factor A: Dysplasia	Factor B: Activity	Interaction term	Comparison of dysplasia and controls amongst inactive UC		Comparison of dysplasia and controls amongst active UC	
	p-value	p-value	p-value	Fold change	p-value	Fold change	p-value
miR-200b-3p	0.314	0.026	0.017	2.148	0.033	0.719	0.259
miR-451a	>0.5	0.402	0.019	0.661	0.155	1.662	0.054
miR-27b-3p	0.082	0.036	0.025	0.645	0.016	1.062	>0.5
miR-155-5p	>0.5	>0.5	0.089	1.330	0.336	0.678	0.142
miR-19a-3p	0.280	0.057	0.323	1.021	>0.5	1.590	0.132
miR-30b-5p	0.116	0.062	0.347	1.116	>0.5	1.525	0.070
miR-30e-5p	0.405	0.278	0.408	0.999	>0.5	0.715	0.222
miR-21-5p	>0.5	0.092	>0.5	0.977	>0.5	0.895	>0.5

### miRNA in situ hybridization

**Fig 3:**
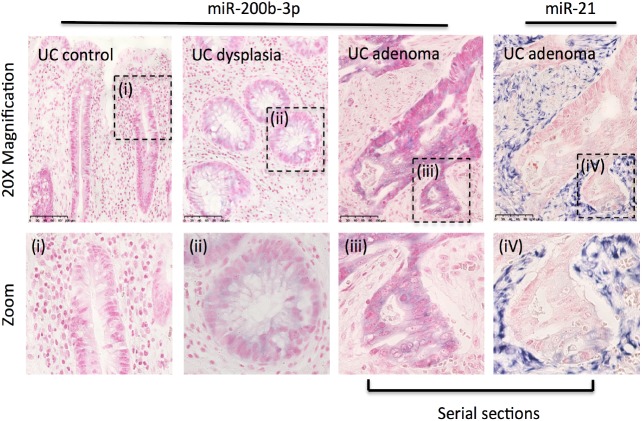
Localisation of miR-200b-3p in ulcerative colitis-dysplasia and adenocarcinomas Representative images of miR-200b-3p staining are shown for UC controls, UC-dysplasia and UC-adenocarcinoma. A pink counterstain (nuclear fast red) is used to visualise the section, and purple staining indicates positive miRNA staining. In a serial section from the UC-adenocarcinoma, miR-21 staining is shown alongside miR-200b-3p to demonstrate the difference in cellular localisation of these miRNA in the gut.

Associations between miR-200b-3p and UC-DYS detected in the array and validation cohort were further investigated using miRNA ISH techniques on archival FFPE blocks. To determine whether miR-200b-3p was linked to CRC progression in UC patients *in situ* analysis was performed on a series of FFPE specimens from UC controls and from UC patients with confirmed UC-DYS and UC-adenocarcinomas; miR-21 levels were analysed in serial sections of a subset of blocks as a positive control for staining.[27] Representative images are shown in Fig 3. Discrete miR-200b-3p staining was identified in the epithelial cells of controls, UC-DYS and UC-adenocarcinomas. In general, the strength of staining increased from UC controls through to UC-adenocarcinomas (Fig 3). By comparison, miR-21 was more robustly expressed and localised to the stroma of UC adenocarcinomas (Fig 3). No positive staining was observed in sections incubated with a scrambled negative control probe.

### Identification of miR-200b-3p targets in UC-dysplasia

By interrogating the gene signatures from cDNA microarray analysis of UC patients with neoplasia (UCN) compared to UC patients,[26] we found 129 mRNAs that were downregulated, corresponding to 76 genes (S4 Table). Since an increase in miRNA levels would result in mRNA downregulation, we investigated whether the reduced expression of these genes is a result of the miRNA action. Among the 76 down-regulated genes, one gene, *INO80D*, was among the targets of miR-200b-3p obtained by miRTarBase (S5 Table), a database of experimentally confirmed miRNA targets. *INO80D* is a regulatory component of the chromatin remodelling INO80 complex, which is involved in transcriptional regulation, DNA replication and probably DNA repair. We expanded our search to target genes that are predicted by bioinformatics algorithms based on the miRNA seed sequence. Five downregulated genes (*INO80D*, *SHROOM1*, *HMBOX1*, *SLC4A4* and *PLEKHA6*) were among the targets predicted bioinformatically by TargetScan (S6 Table), while six genes (*PPARA*, *SHROOM1*, *SMIM5*, *HMBOX1*, *ANK3*, *KMT2C*) were predicted by miRDB (S7 Table). Among these genes, *KMT2C* is a lysine methyltransferase found to be associated with colorectal cancer as well as acute myeloid leukemia.[28,29] KMT2C has also been implicated in gastric cancer and could be useful as a marker of prognosis.[30] Table 4 summarises these results.

**Table 4:** Targets of miR-200b-3p that are downregulated in UC patients with neoplasia compared to UC patients.

Gene	miRNA-target database	Function (Based on Uniprot)
*INO80D*	miRTarBase, TargetScan	Putative regulatory component of the chromatin remodeling INO80 complex which is involved in transcriptional regulation, DNA replication and probably DNA repair.
*SHROOM1*	TargetScan, miRDB	May be involved in the assembly of microtubule arrays during cell elongation.
*HMBOX1*	TargetScan, miRDB	Transcription factor. Isoform 1 acts as a transcriptional repressor. Isoform 4 has very low activity as a transcriptional repressor.
*SLC4A4*	TargetScan	Electrogenic sodium/bicarbonate cotransporter with a Na(+):HCO3(-) stoichiometry varying from 1:2 to 1:3. May regulate bicarbonate influx/efflux at the basolateral membrane of cells and regulate intracellular pH.
*PLEKHA6*	TargetScan	Pleckstrin homology domain-containing family A member 6
*PPARA*	miRDB	Ligand-activated transcription factor. Key regulator of lipid metabolism. Functions as transcription activator for the ACOX1 and P450 genes.
*SMIM5*	miRDB	Small Integral Membrane Protein 5
*ANK3*	miRDB	In skeletal muscle, required for costamere localization of DMD and betaDAG1 (By similarity). Membrane-cytoskeleton linker. May participate in the maintenance/targeting of ion channels and cell adhesion molecules at the nodes of Ranvier and axonal initial segments. Regulates KCNA1 channel activity in function of dietary Mg(2+) levels, and thereby contributes to the regulation of renal Mg(2+) reabsorption
*KMT2C*	miRDB	Histone methyltransferase. Methylates Lys-4 of histone H3. H3 Lys-4 methylation represents a specific tag for epigenetic transcriptional activation. Central component of the MLL2/3 complex, a coactivator complex of nuclear receptors, involved in transcriptional coactivation. KMT2C/MLL3 may be a catalytic subunit of this complex. May be involved in leukemogenesis and developmental disorder

### Analysis of serum miRNA profiles in dysplasia and controls

**Fig 4:**
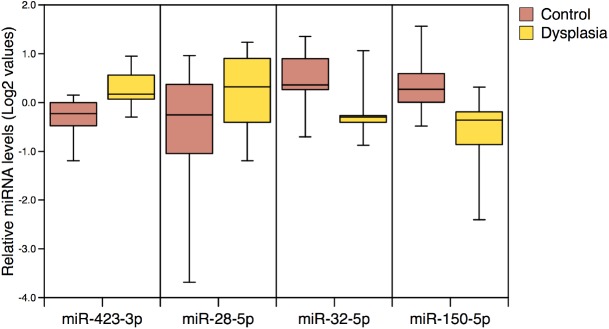
Serum miRNA levels in UC dysplasia and UC controls. Serum levels of miRNAs with a fold change >1.5 in UC patients with dysplasia compared to UC controls are shown. Data are presented as a box and whiskers plot. Differences in the levels of these miRNAs in serum did not reach the statistical significance threshold.

MiRNAs are also present in the serum, where there is the potential to exploit them as non-invasive biomarkers of dysplasia. Hence, we sought to determine serum miRNA levels in UC-DYS patients (n = 8) and UC controls (n = 10). Levels of miRNA in the serum were assessed by qPCR array (Exiqon) and the normalised data were analysed using SAM software as per the tissue arrays (with a cut-off of a fold change>2 set). However, no significantly altered miRNAs in the serum of UC-DYS patients relative to UC controls were identified; with an adjusted cut-off of a fold change>1.5 there were 4 miRNAs were identified: miR-423-3p and miR-28-5p were present at higher levels, whereas serum levels of miR-150-5p, miR-32-5p were reduced in UC-DYS (Fig 4). However, with a false discovery rate of 1.12 a cut-off of a fold change> 1.5 is not stringent enough, and these miRNAs are highly likely to represent false positives.

## Discussion

In this study miRNA profiles associated with dysplastic lesions in UC patients were investigated. Multivariate analysis demonstrated good separation of UC-DYS from UC controls, indicating a shift in miRNA expression profiles in tissue from UC-DYS. However, there was a larger degree of variance between the UC-DYS samples with respect to their miRNA profiles, highlighting the inherent heterogeneity of this group. For future studies it will be important to understand the significance of the profile heterogeneity for UC-DYS progression, as not all LGD will progress to HGD and/or CRC [9–15]. Whether this heterogeneity in miRNA profiles could be exploited to identify the dysplastic lesion most likely to progress remains to be seen. Encouragingly, from a molecular biomarker perspective, the observed changes in miRNA expression in this study appeared specific to UC-DYS because similar changes were not observed in UC-IPs. In fact, the miRNA profiles of UC-IPs were largely indistinguishable from UC controls, suggesting that these lesions might not have pre-malignant potential that could be linked to miRNAs expression changes.

A number of differentially expressed miRNAs in UC-DYS that were identified in this study have been previously linked to IBD-dysplasia,[20] providing independent validation of our results. For example, Olaru and colleagues demonstrated broad increases in the miR-200 family in IBD-dysplasia (both UC and CD patients) relative to chronically inflamed controls, a finding that is in keeping with the up-regulation of miR-200b-3p reported here. While both studies show that increased miR-200b-3p expression is a feature of UC-DYS, it is important to note that in our study, miR-200b-3p was only upregulated in dysplastic lesions of patients in clinical and histological remission, i.e. no inflammatory infiltrates. Inflammation supressed miR-200b-3p and so in patients with ‘active’ disease there was no difference in miR-200b-3p in our validation cohort. Surveillance colonoscopies are already indicated for patients in clinical remission, our data would now indicate that the presence of inflammatory infiltrates in the mucosa should also be considered when assessing the utility as a biomarker. The observation that miR-200b-3p is supressed in patients with evidence of active disease is consistent with reports that show a reduction in miR-200b in inflamed UC colon and reports that the reduced expression of miR-200b in IBD patients is linked to epithelial mesenchymal transition (EMT). [31,32] The effect of inflammation may be to down-regulate miR-200b-3p in other cell types within the mucosa.

As this study focuses predominantly on the analysis of low-grade UC-DYS, it seems likely that the observed up-regulation of the miR-200 family is a very early event in the UC-CRC development pathway. The analysis of miR-200b-3p using miRNA-ISH techniques further supports a functional role for this miRNA in development of UC-CRC as miR-200-3p is localised to intestinal epithelial cells in UC patients consistent with its known role in determining epithelial cell fate.[18,33] Notably, the strength of miR-200b-3p staining increased at each stage of malignancy, UC-DYS and UC-CRC. In line with the findings of this study, increased expression of miR-200b-3p in colon cancer cells *in vitro* has previously been reported,[34] and high expression of miR-200b-3p observed in sporadic colorectal tumours compared to adjacent mucosa.[35]

Previous studies have identified altered mRNA signatures associated with dysplasia in UC that were progressively altered during neoplastic progression.[26] In this study we have utilised this available data set and interrogated it for down-regulated genes with a miR-200b-3p binding site. This list included genes associated with chromatin remodelling and DNA transcription (e.g. INO80D and KMT2C). The functional significance of the suppression of these genes on the progression of UC-DYS is currently unknown. Little is known about the role of the INO80D subunit. However, loss of function mutations in KMT2C, a histone lysine methyltransferase, have been identified in a number of cancers,[36] and colorectal cancer cell lines,[37] pointing towards a tumour suppressor role. This is supported by the fact that KMT2C knockout mouse model that developed ureter epithelial tumors.[38] Correlating miRNA and mRNA expression data sets can give useful insights into the potential action of a miRNA *in vivo* and provide direction for future experimentation that can confirm whether, for example, the suppression of 200b-3p targets in UC-DYS is a direct consequence of the up-regulation of miR-200b-3p.

While there was good concordance between our study and the previous studies for IBD-dysplasia and miR-200b-3p,[19] we were unable to fully validate the increase in miR-21-5p in UC-DYS that was observed in the array and has previously been reported by Ludwig and colleges;[18] instead in the validation analysis this miRNA was much more strongly associated with active inflammation as opposed to the presence of dysplasia, which is known to be an independent risk factor for the development of UC DYS and UC CRC.[39] The link between high miR-21-5p may therefore be highly dependent on the patients inflammatory status. Nevertheless, high levels of miR-21 were observed using miRNA ISH techniques in UC-CRC; in these tumours miR-21 was localised to the stroma and not epithelial cells, a finding which contrasts with Ludwig et al,[18] but accords with Hedbäck et al.[40] The conflicting results between studies for miR-21 localisation is likely a results of using different *in situ* hybridization probes, this will need to resolved before a definitive answer on miR-21 localisation can be given.

Unfortunately, reported differences in miR-31 in IBD-dysplasia,[19] could not be fully interrogated because of its low expression levels in the samples analysed in this study. However, the only two samples from the array where miR-31 was detected above the background threshold levels were dysplastic, which is consistent with the earlier reported increase in dysplasia in the literature. Our data suggest that up-regulation of miR-31 is not sufficiently consistent across UC-DYS specimens for this miRNA to be a good biomarker candidate of LGD, as the absence of its expression could lead to false negative diagnoses of dysplasia.

Attempts to identify a non-invasive serum biomarker of UC-DYS were also made, but no significant differences in serum miRNA expression were observed. This was a novel analysis, and the lack of statistical differences likely reflects the fact that any serum dysplasia signal will be very weak given only a small proportion of cells in the gut are affected, and the serum miRNA pool is derived from all cells in the body. Also, the current study may have been underpowered to detect small differences in miRNA levels in the serum of UC-DYS patients. However, it should be acknowledged that the rarity of the UC-DYS makes large population biomarker studies extremely challenging. Nevertheless, we believe that the data from this study can be used to inform future studies.

There are some limitations to our study. The estimated prevalence of LGD in UC is 15 cases per 1000 patients per year in the UK. [41] Therefore significant numbers of patients need to be screened to identify dysplasia specimens for analysis. To address this we recruited from ten NHS trusts with major inflammatory bowel disease surveillance centres across the UK. Despite this, a clear limitation of this study is the sample size, which reflects the low frequency of UC-DYS, and the difficulty in obtaining sufficient specimens from well-phenotyped patients. Independent validation is therefore an important step that requires adequate numbers. It is therefore encouraging that several miRNAs identified here had been previously linked to IBD-dysplasia, e.g. miR-200b-3p.[19] For future studies a standardized miRNA analysis pipeline should be implemented to facilitate direct cross-comparisons between studies, enabling pooling of data and large population studies, as we have recommended in a recent review;[16] each of the studies performed to date have used different miRNA profiling platforms and methods (Table 5). The data from this study also highlight the need to control for heterogeneity within the UC-DYS group, as potential confounding variables include the size of the field of dysplasia, the extent of inflammation within a lesion, length of disease duration and patient’s medications. Given the relatively advanced age of the UC-dysplasia cohort (median 73-years), there is an increased risk of sporadic CRC and neoplasias, which may not be directly attributed to the underlying presence of UC. These confounders are inherently difficult to control for in small populations, although this is essential if more accurate biomarkers of progression are to be developed, as dysplasia is not a single homogenous group.

**Table 5:** Comparison of miRNA studies in IBD dysplasia.

Study Reference	Study size	Detection methods	Tissue type	Comparisons made
Lewis *et al*	UC controls & UC dysplasia (n = 10 and n = 7, respectively).	Untargetd microNOME-wide analysis: miRCURY LNA™ microRNA Array (7th Gen), Exiqon	Fresh frozen	UC control (active & inactive) Vs. UC dysplasia
Olaru *et al*	Inflamed IBD controls & IBD dysplasia (n = 8, respectively)	Untargetd microNOME-wide analysis: human miR Microarray, Agilent	Fresh frozen	Chronically active IBD Vs. IBD dysplasia
Ludwig *et al*		Target analysis of miR-21: NCodeTM miRNA qRT-PCR method, Invitrogen	FFPE- tissue	Active UC Vs. UC dysplasi & Active CD Vs. CD dysplasia

In summary, CRC is a life-threatening complication of UC, and surveillance programmes use regular colonoscopy to identify early pre-cancerous lesions (dysplasia) and guide patient care. However, we know very little about the molecular changes that occur in these dysplastic lesions and rates of CRC occurrence in patients with confirmed LGD vary widely. A better understanding of the underlying mechanisms of dysplasia may allow for more accurate identification of the lesions that harbour the highest risk of malignancy. In this study we have shown that dysplasia is associated with alterations in the miRNA expression profiles and have demonstrated localisation of this miRNA to epithelial cells in dysplastic lesions and in UC associated CRCs. This study highlights increased expression of miR-200b-3p as a critical component of UC-DYS and a relevant target for therapy.

## Supporting information

S1 Table Table of qPCR primers. (DOCX)

S2 Table List of ulcerative colitis Inflammatory polyps analysed. (DOCX)

S3 Table qPCR analysis of miRNA levels in ulcerative colitis controls and dysplasia. (DOCX)

S4 Table Downregulated mRNAs in UC patients with neoplasia (UCN) compared to UC patients. (XLSX)

S5 Table Target genes of miR-200b-3p in humans obtained by miRTarBase Release 6.0. (XLSX)

S6 Table Target genes of miR-200b-3p predicted by TargetScanHuman Release 6.2. (XLSX)

S7 Table Target genes of miR-200b-3p in humans as predicted by miRDB. (XLSX)

S1 Fig miRNA detection call rate. (DOCX)

S2 Fig Levels of miR-21 and miR-31 in ulcerative colitis controls and dysplasia. (DOCX)

## Abbreviations

ANOVA: analysis of variance
CRC: colorectal rectal cancer
CD: Crohn’s disease
FDR: false discovery rate
FFPE: formalin fixed paraffin embedded
HGD: high grade dysplasia
IBD: inflammatory bowel diseases
UC-IP: post inflammatory polyps
ISH: *in situ* hybridization
LNA: locked nucleic acid
LGD: low grade dysplasia
miRNAs: microRNAs
PCA: principal component analysis
UC: Ulcerative colitis
UC-CRC: UC associated CRC
UC-DYS: UC-Dysplasia
