# Supplementary material for: The miR-200 family is increased in dysplastic lesions in ulcerative colitis patients
Source: PLoS One. 2017 Mar 13;12(3):e0173664. doi: 10.1371/journal.pone.0173664 (PMC5348010; doi:10.1371/journal.pone.0173664)
Supplement: S1 Table — (DOCX) [file pone.0173664.s001.docx]

**S1 Table: Table of qPCR primers.**

| Forward primer assay | Primer assay ID |
| --- | --- |
| hsa-miR-30b-5p | MS00003276 |
| hsa-miR-30e-5p | MS00009401 |
| hsa-miR-21-5p | MS00009079 |
| hsa-miR-19a-3p | MS00003192 |
| hsa-miR-155-5p | MS00034186 |
| hsa-miR-141-5p | MS00008680 |
| hsa-miR-200b-3p | MS00009016 |
| hsa-miR-27b-3p | MS00031668 |
| hsa-miR-451a | MS00004242 |
| Hs-RNU6-2 | MS00033740 |
| Hs-SNORD42b | MS00055090 |
