# Supplementary material for: The miR-200 family is increased in dysplastic lesions in ulcerative colitis patients
Source: PLoS One. 2017 Mar 13;12(3):e0173664. doi: 10.1371/journal.pone.0173664 (PMC5348010; doi:10.1371/journal.pone.0173664)
Supplement: S2 Table — (DOCX) [file pone.0173664.s002.docx]

**S2 Table: List of ulcerative colitis Inflammatory polyps analysed**

| **Pt** | **Gender** | **Age** | **Ethnicity** | **Disease duration** | **Disease extension (Montreal)** | **Smoking habit** | **PP site** | **Active (Y/N)** | **Oral 5ASA** | **Tissue analysed by array** |
| --- | --- | --- | --- | --- | --- | --- | --- | --- | --- | --- |
|  |  |  |  |  |  |  |  |  | **(Y/N)** |  |
| WL017 | M | 28 | Asian | 16 | E3 | Never | Ascending | N | Y | Yes |
| CG008 | M | 49 | White | 28 | E2 | --- | Sigmoid | N | N | Yes |
| PM011 | F | 62 | White | 45 | E3 | Former | All segments | N | Y | Yes |
| PM001 | M | 53 | White | 22 | E3 | Never | Transv | Y | N | Yes |
| WL018 | F | 61 | White | 23 | E3 | Never | Sigmoid | N | N | Yes |
| WL027 | M | 54 | Anglo-Indian | 30 | E3 | Never | Sigmoid | Y | Y | Yes |
| WL014 | M | 74 | White | 15 | E2 | Former | Sigmoid | Y | Y | Yes |
