# Supplementary material for: The miR-200 family is increased in dysplastic lesions in ulcerative colitis patients
Source: PLoS One. 2017 Mar 13;12(3):e0173664. doi: 10.1371/journal.pone.0173664 (PMC5348010; doi:10.1371/journal.pone.0173664)
Supplement: S3 Table — (DOCX) [file pone.0173664.s003.docx]

**S3 Table: qPCR analysis of miRNA levels in ulcerative colitis controls and dysplasia**

| miRNA ID | One-tailed t-test | |
| --- | --- | --- |
|  | Fold change | p-value |
| miR-30b-5p | 1.283 | 0.082 |
| miR-19a-3p | 1.247 | 0.179 |
| miR-200b-3p | 1.119 | 0.323 |
| miR-451a | 1.114 | 0.293 |
| miR-21-5p | 0.972 | 0.455 |
| miR-155-5p | 0.917 | 0.329 |
| miR-27b-3p | 0.868 | 0.117 |
| miR-30e-5p | 0.813 | 0.148 |
