# Supplementary material for: The miR-200 family is increased in dysplastic lesions in ulcerative colitis patients
Source: PLoS One. 2017 Mar 13;12(3):e0173664. doi: 10.1371/journal.pone.0173664 (PMC5348010; doi:10.1371/journal.pone.0173664)

**S1 Figure: miRNA detection call rate.** Plot showing number of microRNAs detectable above background threshold for each sample. The sample highlighted by a red circle was active UC-control biopsy from the caecum and it was excluded due to a lower then average call rate.


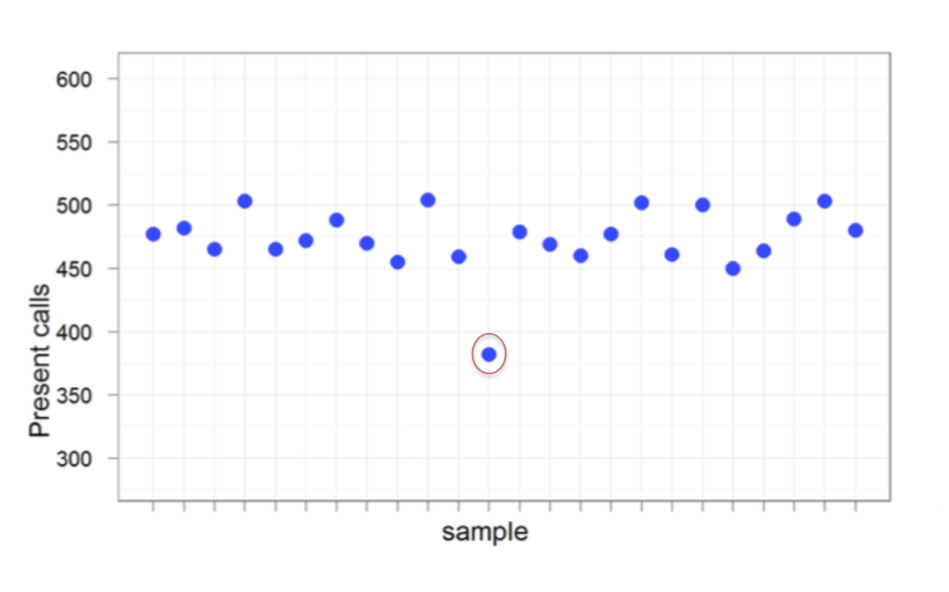

Supplement: S1 Fig — (DOCX) [file pone.0173664.s008.docx]
