# Supplementary material for: The miR-200 family is increased in dysplastic lesions in ulcerative colitis patients
Source: PLoS One. 2017 Mar 13;12(3):e0173664. doi: 10.1371/journal.pone.0173664 (PMC5348010; doi:10.1371/journal.pone.0173664)

**S2 Figure: Levels of miR-21 and miR-31 in ulcerative colitis controls and dysplasia.** Box-plot illustrating expression Levels of miR-21 (A-B), and miR-31 (C-D), 3p and 5p isoforms, respectively. Expression was determined by miRCURY LNA™ microRNA Array (7th Gen), and Log2 normalised data are presented.


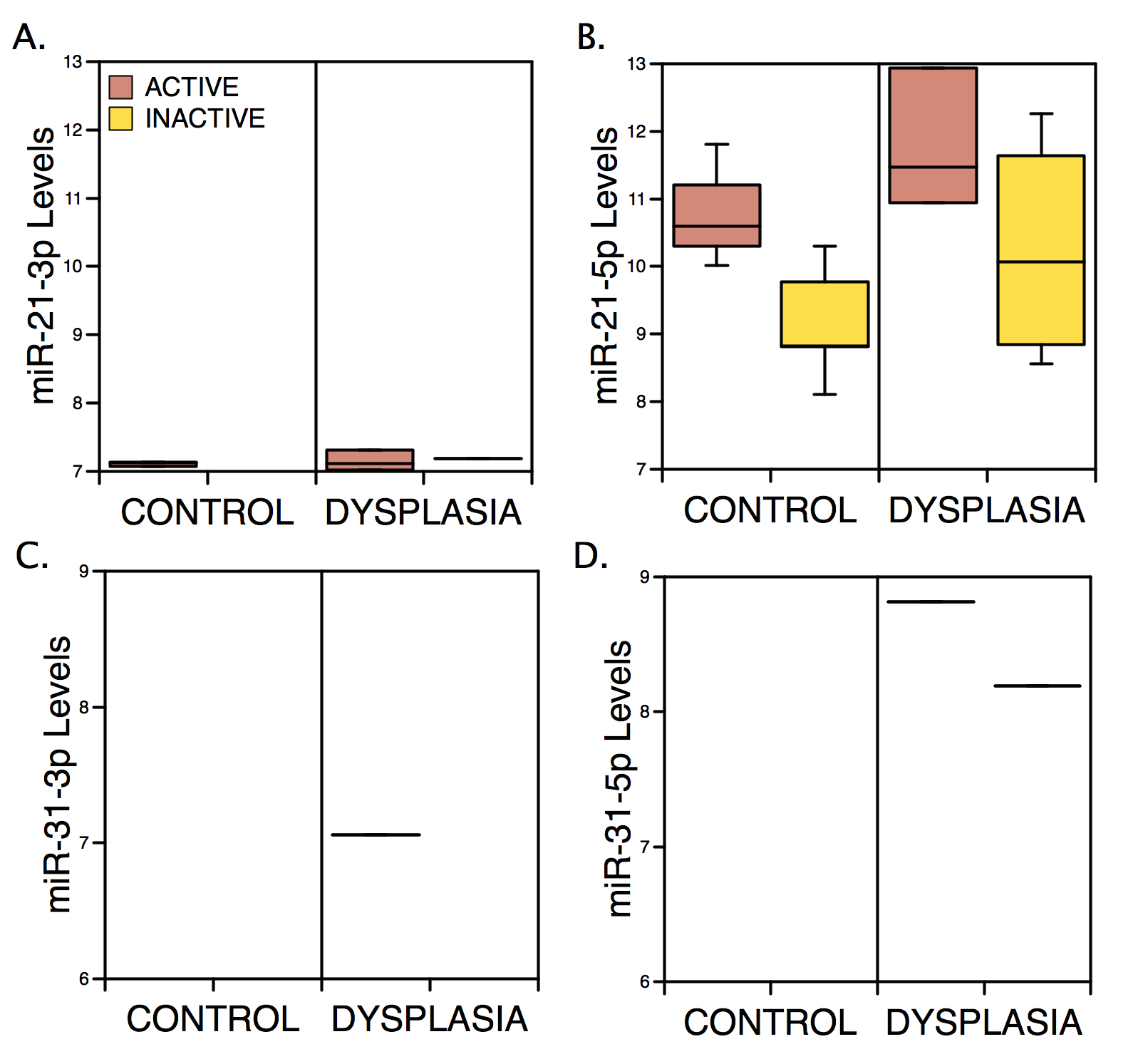

Supplement: S2 Fig — (DOCX) [file pone.0173664.s009.docx]
